# Effect of polymer concentration on the morphology of the PHPMAA-*g*-PLA graft copolymer nanoparticles produced by microfluidics nanoprecipitation†

**DOI:** 10.1039/d3na01038d

**Published:** 2024-03-11

**Authors:** Svetlana Lukáš Petrova, Ewa Pavlova, Václav Pokorný, Vladimir Sincari

**Affiliations:** a Institute of Macromolecular Chemistry v.v.i., Academy of Sciences of the Czech Republic Heyrovsky Sq. 2 162 06 Prague 6 Czech Republic petrova@imc.cas.cz

## Abstract

Successful generation of micelles, vesicles, and/or worms with controllable sizes was achieved through the self-assembly process of the poly[*N*-(2-hydroxypropyl)]methacrylamide-*g*-polylactide (PHPMAA-*g*-PLA) graft copolymer within a microfluidic channel. A product diagram was created to illustrate various morphologies associated with different polymer concentrations, all while maintaining a constant flow velocity ratio between water and the polymer solution.

## Abstract 2

Product diagram shows micelles, vesicles, and/or worms at various PHPMAA-*g*-PLA polymer concentrations. Consistent flow velocity ratios in a microfluidic channel enable successful generation.

## Graphical abstract

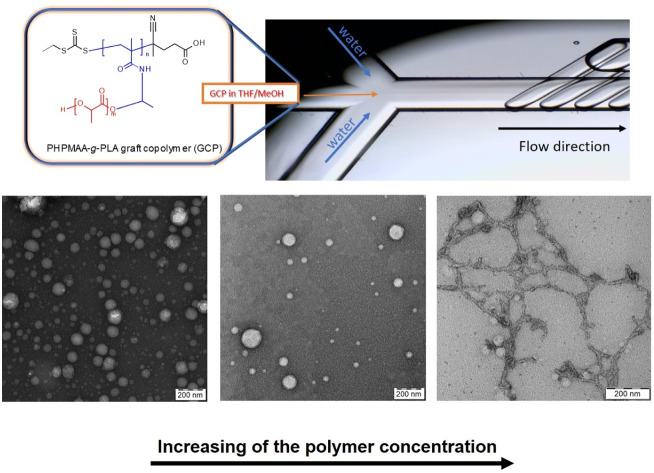

Microfluidic-assisted (MF) synthesis of polymer particles is an advanced and highly precise technique used in the field of materials science and chemistry.^1^ This method utilizes microfluidic devices to control and manipulate the preparation of polymer particles on a small scale, typically at the micro- and even nanoscale levels.^2–5^ It has gained significant attention and popularity due to its ability to produce uniform, monodisperse particles with tailored properties for various applications for targeting therapy,^6^ drug delivery^7^ and chemical material synthesis.^8–10^ The MF method allows for precise control over fluid flow, mixing, and reactions, which is crucial for the synthesis of polymer particles.^11–13^ One of the major advantages of MF is its ability to provide precise control over reaction parameters such as temperature,^14–16^ concentration,^17,18^ and flow rates.^19,20^ This control enables the synthesis of particles with a high degree of uniformity and reproducibility.^11,21,22^ For example, Jiang *et al.* reported the fine-tuning of block copolymer (BCP) self-assembly through the controlled rate of water addition. They showed that a thermodynamically driven formation pathway for polymeric vesicles could be achieved by employing a slow water addition rate of 0.2 wt% per day.^23^ It has been shown in the microfluidic-assisted literature that flow rate ratio and the polymer concentration has a significant effect on the final size, shape and distribution of the self-assembled nanostructures (NPs).^24^ Moreover, achieving precise control over these parameters required the delicate adjustment of kinetic factors.^17^ The self-assembly of amphiphilic copolymers in MF chips into nano-objects with a variety of morphologies is a fascinating area of research at the intersection of polymer science and microfluidics.^17,25^ This process involves the controlled manipulation of these copolymers within microchannels to induce self-organization, resulting in the nano-formation with different shapes and structures. The influence of initial polymer concentration (*C*_initial_) on nanoparticle (NP) morphology can be profound and is a crucial factor in NP synthesis and design. It's worth noting that the specific impact of polymer concentration on NP morphology can vary depending on the particular materials and synthesis methods employed.^17,26^ Researchers often need to optimize the polymer concentration to achieve the desired NP characteristics for their particular application.^1,22^ Optimal polymer concentration can lead to more uniform variation, both in terms of size and shape. This is crucial for applications requiring precise control over NP characteristics. For instance, lower concentrations may favor the formation of spherical nanoparticles (NPs), while higher concentrations might promote anisotropic shapes such as rods or nanowires. Creating highly ordered hierarchical assemblies through kinetic control in microfluidic chips has been a significant challenge until now.

Here, we investigate various nanoparticle morphologies generated *via* microfluidic channels using a biocompatible and biodegradable amphiphilic graft copolymer (AmGCP) with a complex architecture based on poly[*N*-(2-hydroxypropyl)]methacrylamide (PHPMAA) and polylactide (PLA). To the best of our knowledge, this is the first report demonstrating precise control of kinetic processing by adjusting the final polymer concentration (*C*_final_), leading to the self-assembly of AmGCP into micelle-like spheres (Ms), vesicles (Vs), and worms (Ws). It should be noted that there is a paucity of information on the potential impact of *C*_final_ on the morphology of nano-objects while maintaining the same flow rate ratio (*R*) and total flow rate (*V*_total_). This finding provides an additional avenue to refine assembly structures, which is essential, as the size and shape of the assemblies significantly influence their properties and, consequently, impact various applications, mainly in medicine and pharmacy.

Herein, Ms, Vs and Ws were produced using the microfluidic device setup by Dolomite Mitos P-Pumps (Royston, United Kingdom) and equipped with a glass micromixer chip with 12 mixing stage micro-channels of 50 μm × 125 μm (depth × width) (see Fig. S1, in ESI†). The PHPMAA-*g*-PLA graft copolymer was prepared *via* metal-free one-pot/simultaneous RAFT/ROP polymerization strategy similar to the recently published synthetic protocols,^27,28^, (Scheme S1, in ESI†). The synthesis was performed with a feed ratio [HPMAA] : [LA] : [CTA-COOH] : [AIBN] : [DMAP] = [104] : [35] : [1] : [0.25] : [0.25]. The SEC chromatogram (Fig. S2, in ESI†) of the graft copolymer clearly shows that the obtained curve is monomodal and exhibits no competitive side reactions. Notably, the molecular weight distribution (*Đ*) was relatively narrow which indicates that the combination of ROP and RAFT polymerization in a one-pot/one-step protocol proceeded as a living process and the obtained graft copolymer has a controlled structure (as determined by DMF SEC, *M*_n_ = 21 900 g mol^−1^, *Đ* = 1.13; *vs.* poly(methyl methacrylate) calibration standards). The PHPMAA_104_-*g*-PLA_35_ GCP (where the numbers in the subscripts indicate the number of repeat units of the blocks), was dissolved in THF/MeOH (80/20) (v/v) to achieve *C*_final_ of 5.0, 10.0 and 20.0 mg ml^−1^. These solutions were pumped through the middle channel, while MilliQ water was concurrently pumped through the side channels using two separate liquid streams controlled *via* computer software. The flow rate of the water phase (WP) was a variable parameter (200, 300, and/or 500 μL min^−1^), while the flow rate of the organic phase (OP) was held constant (100 μL min^−1^) until the desired WP-to-OP ratio was attained or it was (100 μL min^−1^/300 μL min^−1^). After evaporating the organic solvent using a rotary vacuum evaporator, polymer colloids were obtained. The resulting self-assemblies were characterized in detail using dynamic light scattering (DLS), transmission electron microscopy (TEM), and small-angle X-ray scattering (SAXS) techniques. The size, morphology, and polydispersity of the self-assembled nanostructures were precisely controlled through the MF-assisted method. Well-defined Ms, Vs, and/or Ws were achieved by employing microfluidic chip with a micromixing architecture (see Fig. S1, in ESI†). Another example showcasing a range of morphologies, including Vs, interconnected worm-like micelles, and spherical micelles Ms, can be found in the work of Lecommandoux *et al.*^25^ They explored the self-assembly of amphiphilic hybrid diblocks using a combination of microfluidic chip systems and more conventional techniques such as dialysis or direct dissolution.

To successfully dissolve the PHPMAA_104_-*g*-PLA_35_ GCP, an organic solvent mixture needed to be optimized in the first stage. We have selected THF/MeOH 80/20 v/v as the organic phase and water as the aqueous phase. Systematically investigation on the effect of the *C*_final_ ranging from 0.5, 1.0 to 2.0 wt%, on the formation of NPs with constant flow velocity ratio *R*. The self-assembly process in all experiments were conducted using a *C*_initial_ of the copolymer solution 5.0 mg ml^−1^, which is the highest concentration that prevents the formation of macroscopic aggregates within the microfluidic chip.

Fig. 1 depicts TEM images and a color scheme illustrating the progression of polymer nanoparticle morphology at different *C*_final_ values. In the schematic representation, the blue color denotes the hydrophilic segment of the GCP, PHPMAA, while the red color signifies the hydrophobic PLA block. Notably, the green shade, particularly evident at *C*_final_ = 1.0 wt%, indicates the presence of water trapped inside the vesicle.

**Fig. 1:**
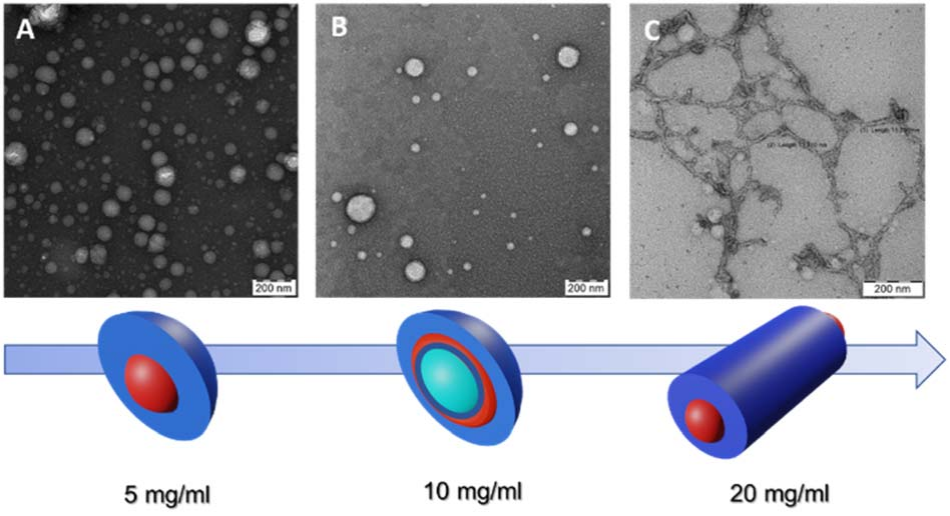
TEM images and schematic representation of self-assembled PHPMA-*g*-PLA at different *C*_final_: (A) Ms at 0.5 wt%, (B) Vs at 1.0 wt% and (C) Ws at 2.0 wt%.

The use of the lowest *C*_final_ (0.5 wt%) resulted in the formation of micelle-like spherical nano-objects. Indeed, the TEM illustration clearly demonstrates the presence of well-defined spherically assembled micelles with a particle diameter of approximately 100 to 130 nm see, Fig. 1A (a zoomed TEM image is provided in the ESI, Fig. S3A†). Furthermore, the mean hydrodynamic diameter (*D*_H_) and the polydispersity index (PDI) of the obtained micelle-like spherical nano-objects were determined by DLS. The analysis revealed monomodal particles size distribution, with a *D*_H_ of 126 nm and a relatively narrow polydispersity of 0.13 (see, Fig. S4, in ESI,†Table 1). In this case, DLS analysis shows an unexpectedly high *D*_H_, deviating from the expected characteristics of micelles. Instead, the *D*_H_ value closely resembles that typically reported for vesicle systems. The purported reason for identical DLS values could be attributed to the correlation between the mean sphere diameter (*D*) and the degree of polymerization (DP) of the core-forming block.^29–31^ Moreover, the *D*_H_ of NPs in a good solvent, assessable through DLS,^32^ typically exceeds that of core NPs due to polymer brush swelling and grafted chain polydispersity.^33,34^

**Table 1:** Physicochemical characteristics of the PHPMAA-*g*-PLA NPs at different *C*_final_

*C*_final_	*D*_H_/nm, (PDI)^a^	Morph.^b^	*D*/nm^c^
0.5 wt%	126 (0.13)	Ms	100–130
1.0 wt%	25–60/124 (0.21)	Vs/Ms	105–135
2.0 wt%	140 (0.34)	Ws	15 ± 3.2

a Hydrodynamic diameter and dispersion from DLS.

b Nanoparticle morphology from TEM (Ms = micelles, Vs = vesicles and Ws = worms).

c Particle diameter from TEM.

Our research delves deeper into the nanoprecipitation process of the PHPMAA_104_-*g*-PLA_35_ GCP solution within MF chip, specifically at a concentration of 1.0 wt% as the *C*_final_. As shown in Fig. 1B, of PHPMAA_104_-*g*-PLA_35_ nano-objects, a predominant vesicular phase is observed, characterized by diameters ranging from 105 to 135 nm. Moreover, the TEM image reveals the presence of a small population of micelles as well, with a particle diameter of about 25–60 nm (Fig. 1B). For a clearer visualization of the Vs shape, refer to the zoomed TEM image in Fig. S3B within the ESI.† Size measurements employing DLS indicate a *D*_H_ of approximately 124 nm, coupled with a relatively broad of 0.21. The obtained results could be accepted as evidence of the presence of Vs/Ms mixtures. The corresponding dynamic light scattering particle size distribution is shown in (Fig. S5, in ESI†), Table 1. These findings are in accordance with the observations from the TEM image, providing a comprehensive understanding of the formed nanostructures.

We finally investigated the use of microfluidic mixing for assembling PHPMAA_104_-*g*-PLA_35_ NPs at the highest *C*_final_ (2.0 wt%). In this instance, the MF-assisted technique resulted in the formation of polydisperse wormlike particles, with an estimated size of approximately (*D*) 15 ± 3.2 nm, as depicted in the TEM micrograph (refer to Fig. 1C). This observation explains the pretty broad particle size distributions (PDI = 0.34) obtained by DLS, with an apparently *D*_H_ around 140 nm, see Table 1 (Fig. S6, in ESI†), Such polydispersity is a common characteristic observed in nano-objects exhibiting a wormlike morphology.^25,30,35^ Recently, Zhu J. and co-workers, demonstrated, that within a microfluidic channel, the self-assembly of polystyrene-*block*-poly(4-vinyl pyridine) leads to the formation of segmented wormlike micelles (SWMs) with adjustable sizes. This approach enables the precise regulation of structured SWMs by varying parameters such as the *R* and the total flow velocity.^17^

Moreover, SAXS measurements were employed as a powerful analytical technique to confirm the presence of different morphologies. Scattering curves for NPs at *C*_final_ 0.5, 1.0, and 2.0 wt% were obtained, as illustrated in Fig. 2. While the low-*q* region with the characteristic power laws for each morphology (*q*^−0^ for Ms, *q*^−2^ for Vs, and *q*^−1^ for Ws)^29^ could not be reached (due to experimental limitations), the initial slope for each curve is noticeably different and the order of these slopes agrees with our findings from the other characterization methods.

**Fig. 2:**
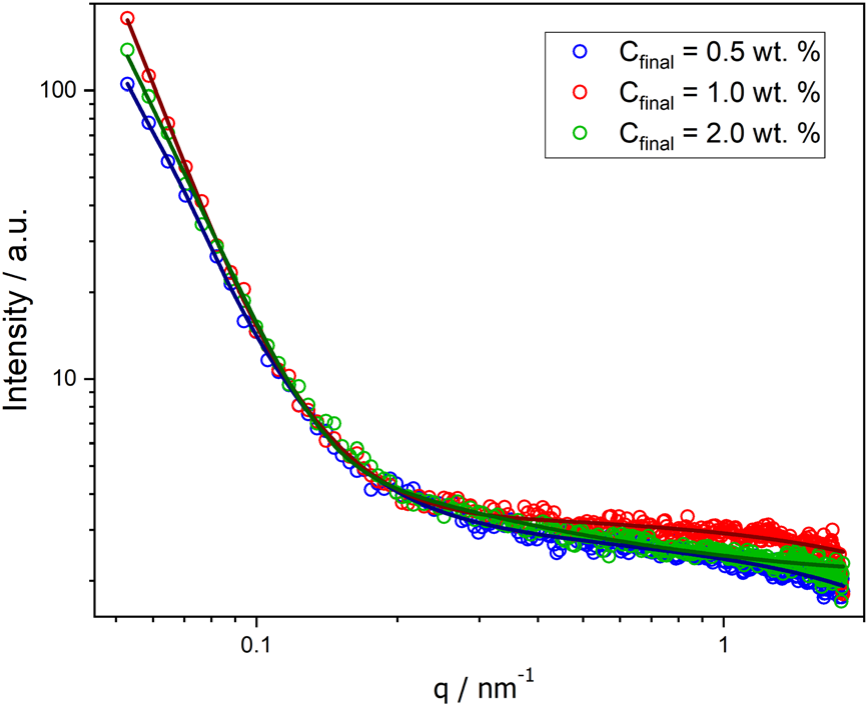
SAXS curves for PHPMA-*g*-PLA NPs formed in microfluidic channels at different *C*_final_: 0.5 wt% (Ms, blue circles), 1.0 wt% (Vs, red circles), and 2.0 wt% (Ws, green circles).

Successful fitting for nano-objects at *C*_final_ 0.5 and 1.0 wt% was achieved using a core–shell sphere model with a log-norm distribution for the outer size. The scattering patterns were modeled by incorporating the form factors of spherical micelles^36^ and vesicles,^37^ complemented by a background model of Gaussian polymer chains.^38^

1 *I*(*q*) = [*K* (*q*, *R*_core_, Δ*η*_core_) + *K* (*q*, *R*_core_ + Δ*R*_shell_, Δ*η*_shell_) − *K* (*q*, *R*_core_, Δ*η*_shell_ − Δ*η*_core_)]^2^ + background

with2
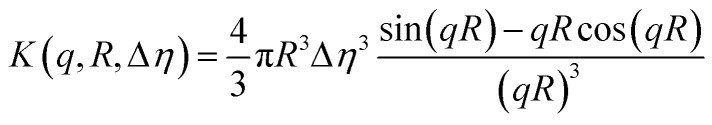
where *R*_core_ is the radius of the core, Δ*R*_shell_ is the shell thickness, and Δ*η* is the scattering length density difference between cor/shell and the solvent. These parameters are summarized in Table 2.

**Table 2:** Parameters of the core–shell model for PHPMAA-*g*-PLA NPs at *C*_final_ 0.5 and 1.0 wt%

*C*_final_	0.5 wt%	1.0 wt%
*R*_core_ [nm]	74.9	52.7
Δ*R*_shell_ [nm]	0.06	16.2
Δ*η*_core_	3.66 × 10^−5^	7.56 × 10^−5^
Δ*η*_shell_	4.16 × 10^−3^	8.30 × 10^−5^

For PHPMAA-*g*-PLA NPs at *C*_final_ 0.5 wt%, the smallest initial slope of the SAXS curve (see Fig. 2, blue circles) and the very small shell thickness (Δ*R*_shell_) value of 0.06 nm both indicate the formation of core–shell spherical aggregates – micelles with the total particle diameter *D* = 2(*R*_core_ + Δ*R*_shell_) of 150.0 nm. In contrast, PHPMAA-*g*-PLA NPs at *C*_final_ 1.0 wt% showed the biggest initial slope of the observed curves (Fig. 2, red circles) and the shell thickness (Δ*R*_shell_) value is relatively large at 16.2 nm, which corresponds with the structure of polymeric vesicles with the total particle diameter *D* of 137.8 nm.

Compared to the other two samples, the SAXS curve of PHPMAA-*g*-PLA NPs at *C*_final_ 2.0 wt% has a different overal and the initial slope is in the middle (see Fig. 2, green circles). This agrees with our observation of worm-like structure shown in Fig. 1C. To achieve an optimal fit to the SAXS pattern, a combination of models was utilized. Specifically, the WormLikeChainEXV model, as outlined by Pedersen *et al.*, was employed to characterize the wormlike chains,^36^ while the extended Guinier law model^39^ was used to describe the corona block. The mean worm contour length or total length (*L*_w_) was determined to be 5592 nm. The mean worm width (*W*_w_) was calculated to be 13 nm, considering the circular cross-section of the worms. This result closely aligns with the estimate obtained from TEM images, where *W*_w_ was measured as 15 ± 3.2 nm. To derive this calculation, the equation *W*_w_ = 2*R*_sw_ + 4*R*_g_ was employed, where *R*_sw_ represents the radius of the worm core cross-section, and *R*_g_ represents the radius of gyration of the corona chains (PHPMAA). Based on the fit to the SAXS pattern, the Kuhn length – the length of two neighboring rigid sections (*RL*_w_) – was found to be approximately 870 nm (with one segment measuring 435 nm). Additionally, the overall diameter *d* of the corona block was determined to be 3472 nm.

In summary, the study focuses on the kinetic-controlled self-assembly of PHPMAA-*g*-PLA graft copolymer within microfluidic chips, presenting a robust and adaptable method for generating NPs with varied morphologies. Notably, the research achieves well-defined nano-objects across different *C*_final_ while keeping the total flow velocity of water and the GCP solution, or their flow velocity ratio, constant. Based on the investigations, we constructed a morphological diagram of nano-objects related to the *C*_final_ as well as the complex architecture of the used GCP, which is crucial for the rational design and fabrication of complex hierarchical GCP nano-objects. This innovative approach holds promise for opening up new avenues in advanced materials, spanning applications from drug delivery to nanotechnology.

## Author contributions

S. L. P. performed synthesis of PHMAA_104_-*g*-PLA_35_ graft copolymer *via* one-pot/one-step ROP/RAFT approach and wrote the manuscript. E. P. performed TEM experiments and analyzed the data and V. P. performed SAXS experiments and analyzed the data. V. S. performed MF, DLS experiments and analyzed the DLS data.

## Conflicts of interest

There are no conflicts to declare.

## Supplementary Material

NA-006-D3NA01038D-s001
