# Supplementary material for: Effect of polymer concentration on the morphology of the PHPMAA-g-PLA graft copolymer nanoparticles produced by microfluidics nanoprecipitation
Source: Nanoscale Adv. 2024 Mar 11;6(8):1992–6. doi: 10.1039/d3na01038d (PMC11019477; doi:10.1039/d3na01038d)
Supplement: NA-006-D3NA01038D-s001 [file NA-006-D3NA01038D-s001.pdf]

Electronic Supplementary Material (ESI) for Nanoscale Advances.
This journal is © The Royal Society of Chemistry 2024

Supplementary Information for the Manuscript Entitled

Effect of Polymer Concentration on the Morphology of the PHPMAA-*g*-PLA Graft Copolymer Nanoparticles Produced by Microfluidics Nanoprecipitation

Svetlana Lukáš Petrova*, Ewa Pavlova, Václav Pokorný and Vladimir Sincari*

Institute of Macromolecular Chemistry v.v.i., Academy of Sciences of the Czech Republic, Heyrovsky Sq. 2, 162 06 Prague 6, Czech Republic

*Correspondence: petrova@imc.cas.cz; sincari@imc.cas.cz

Synthesis of PHPMAA-*g*-PLA graft copolymer *via* one-pot/one-step protocol.

The synthesis of the $PHPMAA_{104}$-*g*-PLA_{35} graft copolymer was realized *via* metal-free one-pot/simultaneous RAFT/ROP polymerization. The protocol for the simultaneous RAFT/ROP synthetic process is thoroughly described in accordance with our recently published studies [1]. The used feed ratio is [HPMAA]:[LA]:[CTA-COOH]:[AIBN]:[DMAP]=[104]:[35]:[1]:-[0.25]:[0.25].

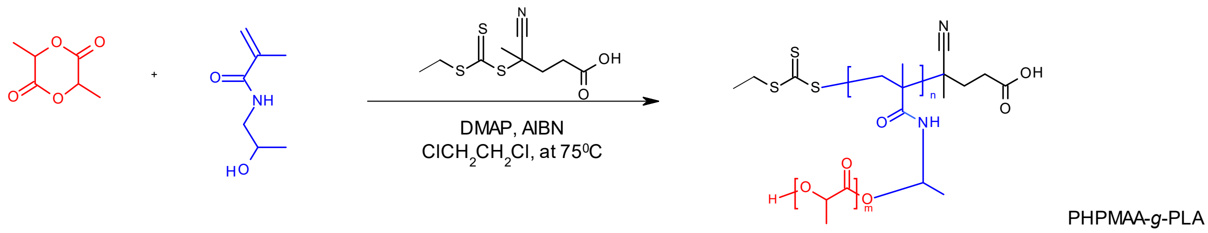

Scheme S1. Synthetic scheme for the synthesis of PHPMAA-*g*-PLA graft copolymers *via* one-pot/ simultaneous ROP/RAFT polymerization.

Hydrodynamic flow focusing nanoprecipitation microfluidics (MF).

Microfluidics is an emerging field for the production of particles, droplets, capsules and multiple emulsions. Among other common techniques such as nanoprecipitation, film-rehydration and electroformation, microfluidics allows more controlled and precise way for the

production of highly monodisperse polymeric structures including micelles, worms and vesicles.

Microfluidics nanoparticle preparation systems with high shear micromixing and hydrodynamic focusing methods give precise particle sizes in the range of ~20 nm to 500 nm. The microfluidics use continuous and controllable laminar flow for the production of high-yield and high-quality nanoparticles. The superior control over the size, shape and morphology of particles enables greater reproducibility and scalability. These substantial improvements in nanoparticle generation are highly valued in pharmaceutical industry compared to conventional batch methods. In this work, the commercial microfluidic chip from Dolomite company (Figure S1) was used for production of well-defined nanosized polymersomes.

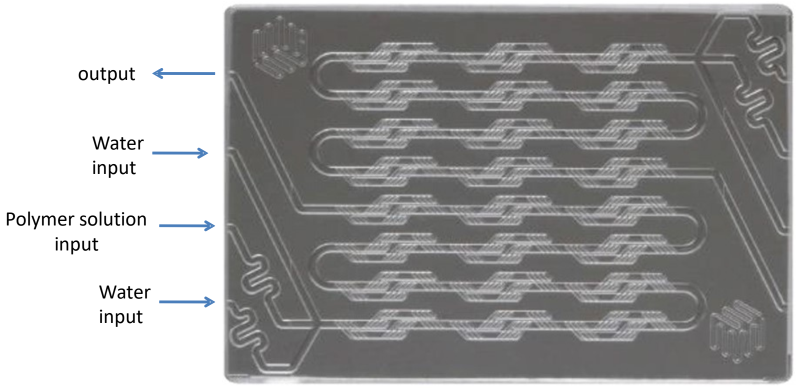

Figure S1. Dolomite setup of the micromixing chip with two water inputs and one polymer solution input.

The mixing time (τ_{mix}) for the hydrodynamic flow focusing using a two-dimensional model is estimated according to equation 1:

$$\tau_{mix} = \frac{w^2}{9D}\frac{1}{(1+1/R)^2} \quad (1)$$

where D is diffusivity of the solvent, w is channel width, and R is the ratio of flow rate of the polymeric stream to the total flow rate of water. For $D \sim 10^{-9}$ $m^2 \cdot s^{-1}$ and $w = 125$ µm equation 1 predicts a mixing time in the range of 0.6 to 7 ms for typical flow ratios ($R = 0.04 - 0.8$) in this device. Particles sizes can be finetuned by changing the flow ratios between the organic and the aqueous phase, the slowest flow gives the smallest particles which was explained by the decrease in the τ_{mix}. Furthermore, besides the lower particle sizes a decrease in the width of the particle size distribution is observed compared with the bulk nanoprecipitation process. The

NPs produced using this microfluidic device always presented size distributions with dispersity below 0.1 when measured by dynamic light scattering.

Instruments and Analyses:

Characterization techniques

Size exclusion chromatography (SEC). The apparent average molecular weight (i.e., number-average molecular weight (M_n) and weight-average molecular weight (M_w) and the dispersity ($Đ$) of the PHPMAA-*g*-PLA copolymer was obtained by SEC performed at 25 ºC with two PLgel MIXED-C columns (300 x 7.5 mm, SDV gel with particle size 5 µm; Polymer Laboratories, USA) and with UV (UVD 305; Watrex, Czech Republic) and RI (RI-101; Shodex, Japan) detectors. N,N-Dimethylformamide (Sigma Aldrich, Czech Republic) with LiBr (0.01 % v/v) was used as mobile phase at flow rate of 1 $mL.min^{-1}$. The M_n values were calculated using Clarity software (Dataapex, Czech Republic). Calibration with poly(methyl methacrylate (PMMA)) standard was used.

The dynamic light scattering (DLS) measurements were performed using an ALV CGE laser goniometer consisting of a 22 mW HeNe linear polarized laser operating at a wavelength (λ = 632.8 nm), an ALV 6010 correlator, and a pair of avalanche photodiodes operating in pseudo-cross-correlation mode. The samples were loaded into 10 mm diameter glass cells and maintained at 37 ± 1 °C. The data were collected using the ALV Correlator Control software and the counting time was 45 s. The measured intensity correlation functions $g_2(t)$ were analyzed using the algorithm REPES (incorporated in the GENDIST program) [2], resulting in the distributions of relaxation times shown in equal area representation as $\tau A(\tau)$. The mean relaxation time or relaxation frequency ($\Gamma = \tau^{-1}$) is related to the diffusion coefficient (D) of the nanoparticles as $D = \frac{\Gamma}{q^2}$ where $q = \frac{4\pi n \sin\frac{\theta}{2}}{\lambda}$ is the scattering vector being n the refractive index of the solvent and θ the scattering angle. The hydrodynamic radius (R_H) or the distributions of R_H were calculated by using the Stokes-Einstein relation:

$$R_H = \frac{k_B T q^2}{6\pi\eta D}\tau \quad (2)$$

being k_B the Boltzmann constant, T the absolute temperature, and η the viscosity of the solvent. The transition temperature (T_{tr}), characterizing the polymer chain conformation changes, was

evaluated from the temperature dependence of the hydrodynamic diameter (D_H) and was determined from the intersection point of two lines formed by the linear regression of a lower horizontal asymptote and a vertical section of the S-shaped curve (sigmoidal curve) fit.

The small-angle X-ray scattering (SAXS) experiments were performed using a pinhole camera (MolMet, Rigaku, Japan, modified by SAXSLAB/Xenocs) attached to a microfocused X-ray beam generator (Rigaku MicroMax 003) operating at 50 kV and 0.6 mA (30 W). The samples were sealed into boro-silicate capillaries (~2 mm diameter) and the scattering intensities were recorded using Pilatus3 R 300 K hybrid photon-counting detector with 1.36 m sample-to-detector distance and exposure time of 480 min. The scattering profiles were fitted using SASFit software. The influence of the solvent, which was water in this case, was approximated using the constant function I_{H2O} = 3.41. The electron densities were determined based on the chemical structure of each polymer block and the mass densities, and the size distribution of the samples was accounted for in the form factors by using a log-normal distribution. For the patterns of particles with C_{final} of 0.5 and 1.0 wt.%, the core-shell model [3] with form factors of spherical core-shell assemblies were used during the fitting procedure. The fitting parameters of this model are radius of the core (R_C), thickness of the shell (t_{shell}), scattering length density difference between the core and the solvent ($\Delta\eta_{core}$) and scattering length density difference between the shell and the solvent ($\Delta\eta_{shell}$). For particles with C_{final} of 2.0 wt.%, a combination of the WormLikeChainEXV model [4] and the extended Guinier law model [5] was used to fit the obtained patterns. The fitted parameters of the WormLikeChainEXV model are Kuhn length (RL_W), contour length (L_w) and radius of the worm core cross-section (R_{SW}). Parameters of the extended Guinier model are the scaling factor (I_0), radius of gyration (R_g), and the dimensionality parameter (a) describing the overall shape.

The transmission electron microscopy (TEM) observations were studied using microscope Tecnai G2 Spirit Twin 120kV (FEI, Czech Republic) operated at an acceleration voltage of 120kV, 2 μL of the aqueous solutions were dropped onto a copper TEM grid (400 mesh) coated with thin, electron-transparent carbon film. The excess of solution was removed out by touching the bottom of the grid with filtering paper. This fast removal of the solution was performed after 1 min of sedimentation in order to minimize oversaturation during the drying process. Nanoparticles were negatively stained with uranyl acetate (2 μL of 2 wt.% solution dropped onto not completely dried grid and removed after 30 s in the same way as described above). The samples were left to dry completely at ambient temperature and then observed in the TEM

microscope. Under these conditions, the micrographs displayed negatively stained background with bright nanoparticles.

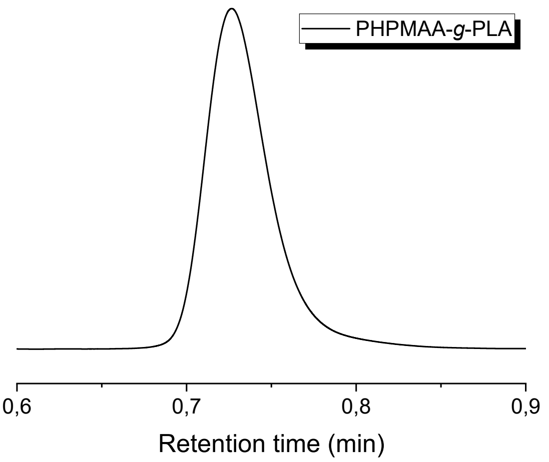

Figure S2. SEC chromatogram in DMF of the PHPMAA-*g*-PLA graft copolymer.

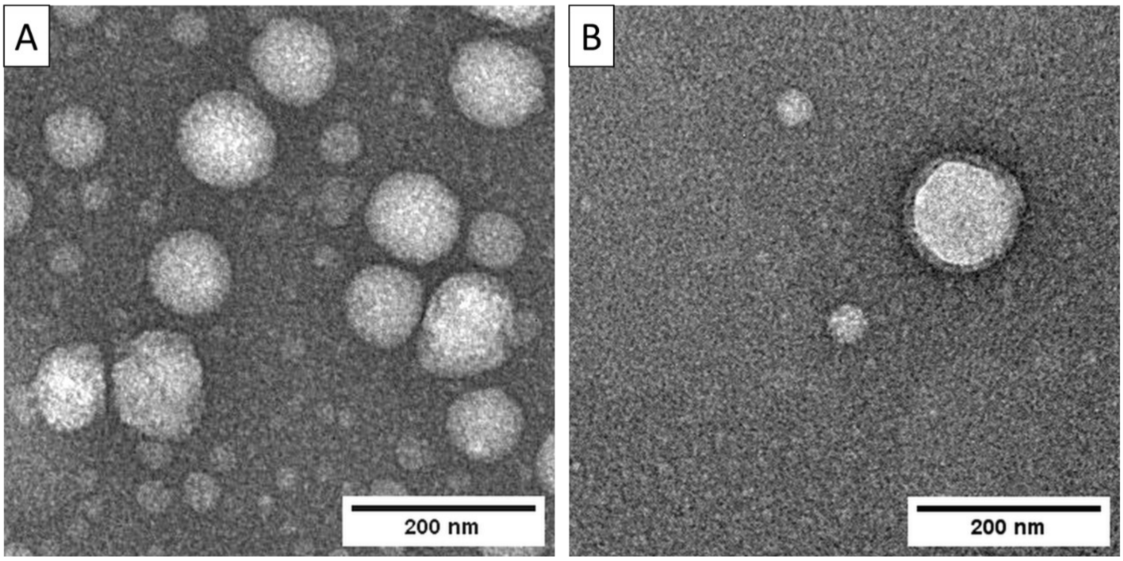

Figure S3. A zoomed TEM images of self-assembled PHPMA-*g*-PLA at different C_{final}: (**A**) Ms at 0.5 wt. %, and (**B**) Vs at 1.0 wt.

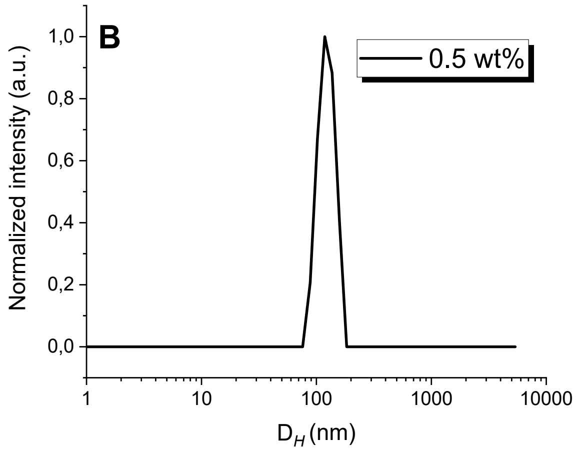

Figure S4. DLS recorded for PHPMAA-*g*-PLA graft copolymer Ms at C_{final} 0.5 wt%.

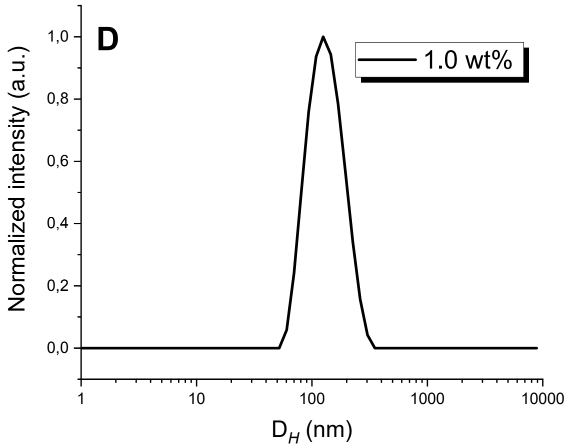

Figure S5. DLS recorded for PHPMAA-*g*-PLA graft copolymer Vs at C_{final} 1.0 wt%.

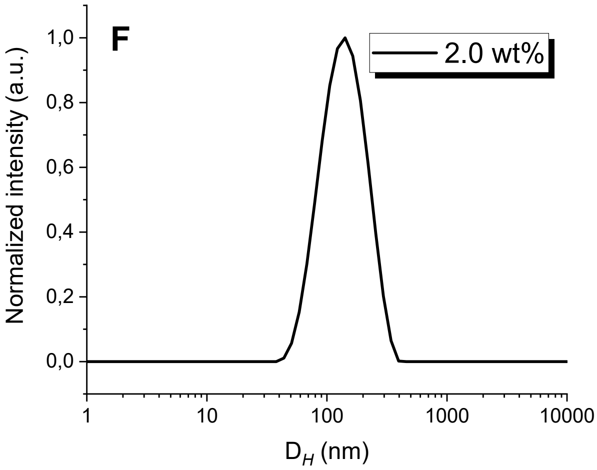

Figure S6. DLS recorded for PHPMAA-*g*-PLA graft copolymer Ws at C_{final} 2.0 wt%.

1 S. Lukáš Petrova, V. Sincari, R. Konefał, E. Pavlova, V. Lobaz, O. Kočková and M. Hrubý, *Macromol. Chem. Phys.*, , DOI:10.1002/macp.202300271.

2 J. Jakeš, *Czechoslov. J. Phys.*, 1988, **38**, 1305–1316.

3 A. Guinier, G. Fournet, C. B. Walker and G. H. Vineyard, *Phys. Today*, 1956, **9**, 38–39.

4 J. S. Pedersen, *J. Appl. Crystallogr.*, 2000, **33**, 637–640.

5 A. Guinier, P. Lorrain, D. S.-M. Lorrain and J. Gillis, *Phys. Today*, 1964, **17**, 70–72.
